# Discriminative Learning for Alzheimer's Disease Diagnosis via Canonical Correlation Analysis and Multimodal Fusion

**DOI:** 10.3389/fnagi.2016.00077

**Published:** 2016-05-17

**Authors:** Baiying Lei, Siping Chen, Dong Ni, Tianfu Wang

**Affiliations:** National-Regional Key Technology Engineering Laboratory for Medical Ultrasound, Guangdong Key Laboratory for Biomedical Measurements and Ultrasound Imaging, School of Biomedical Engineering, Shenzhen UniversityShenzhen, China

**Keywords:** Alzheimer's disease diagnosis, bag of feature, canonical correlation analysis, fusion, normalization

## Abstract

To address the challenging task of diagnosing neurodegenerative brain disease, such as Alzheimer's disease (AD) and mild cognitive impairment (MCI), we propose a novel method using discriminative feature learning and canonical correlation analysis (CCA) in this paper. Specifically, multimodal features and their CCA projections are concatenated together to represent each subject, and hence both individual and shared information of AD disease are captured. A discriminative learning with multilayer feature hierarchy is designed to further improve performance. Also, hybrid representation is proposed to maximally explore data from multiple modalities. A novel normalization method is devised to tackle the intra- and inter-subject variations from the multimodal data. Based on our extensive experiments, our method achieves an accuracy of 96.93% [AD vs. normal control (NC)], 86.57 % (MCI vs. NC), and 82.75% [MCI converter (MCI-C) vs. MCI non-converter (MCI-NC)], respectively, which outperforms the state-of-the-art methods in the literature.

## Introduction

Brain disease or disorder [i.e., Alzheimer's disease (AD), Parkinson's disease] has arisen as a serious social issue in line with aging populations and has garnered great attention over the past decade. As one of the most common and progressive impairment of cognitive dementia, AD, and mild cognitive impairment (MCI) mainly occur in the elderly person over 65 years old. Recently, the Alzheimer's Association (2014) has reported that the number of elderly people with either AD or MCI increases significantly, and hence it is of great importance for early diagnosis and symptomatic treatments of the disease.

Until now, there are a myriad of literature focused on developing computerized methods for the AD/MCI prediction and diagnosis, and great success has been witnessed in numerous modalities such as magnetic resonance imaging (MRI; Davatzikos et al., 2010; Cuingnet et al., 2011; Li et al., 2011; Wee et al., 2011, 2012; Zhang and Shen, 2011; Zhou et al., 2011), positron emission tomography (PET; Nordberg et al., 2010), and functional MRI (fMRI; Greicius et al., 2004). In the literature, there are many methods to diagnose this neurological disease via feature or score fusion (Perrin et al., 2009; Wee et al., 2011; Catana et al., 2012; Westman et al., 2012; Ramirez et al., 2013; Jiang and Lai, 2014; Suk et al., 2014; Zhu et al., 2014a; Lei et al., 2015a). It was shown in previous study that the unimodal data (i.e., MRI or PET) provides limited information for AD diagnosis, whereas data fusion such as MRI and PET substantially boosts the diagnostic accuracy thanks to the complementary information. In addition, it will boost AD/MCI diagnosis performance by fusing scores and features. For instance, in Liu M. et al. (2014), single modality with a hierarchical feature representation was proposed for the AD/MCI diagnosis and achieved high success. However, it is still a challenging issue to incorporate features from different modalities and complex patterns in canonical space for computer-aided disease diagnosis.

Canonical correlation analysis (CCA) has been successfully used to fuse features of different modalities in various applications (Nielsen, 2002; Hardoon et al., 2007; Zhang et al., 2007; Hardoon and Shawe-Taylor, 2011; Sun et al., 2011, 2013; Hou and Sun, 2014; Yeh et al., 2014; Yuan et al., 2014). In this work, we also use CCA for feature fusion with the rationale that CCA can handle the heterogeneous characteristics of the features by transforming them into a canonical common space, so that it becomes easy to construct a robust model of classification. By investigating information not only from individual modalities, but also from the shared features, the learning and classification performance can be further enhanced (Sun et al., 2013). As proved in Shen et al. (2014), auxiliary features boost classification performance in some applications. Consequently, the auxiliary features in a canonical subspace are incorporated for AD disease diagnosis. Since fusion is highly effective for classification task, multi-modal data is fused by CCA. In addition, a novel hybrid level fusion is also designed in our method to enhance diagnosis performance.

State-of-the-art low-level feature representations and patterns include cortical thickness, gray matter/voxel intensity extracted from voxel, patch, or region-of-interest (ROI) are widely used for AD/MCI diagnosis. Both single feature (Tamaki et al., 2013; Stanciu et al., 2014) and feature fusion have commonly used for diagnosis as well. Recently, visual feature such as the densely sampled scale-invariant feature transformation (SIFT; Jégou et al., 2012; Vedaldi and Zisserman, 2012; Cinbis et al., 2013; Gorelick et al., 2013; Sánchez et al., 2013; Lei et al., 2014, 2015b) has become a very popular feature descriptor. However, this low-level visual feature often suffers from noises, whereas high-level or abstract feature is able to withstand the noises to achieve robustness. Hence, low-level visual features are transformed into a high-level representation (e.g., histogram of occurrence).

Large feature dimension and small sample size (i.e., “small-n-large-p” problem) is always a challenging issue to identify clinical subject correctly via robust modeling. Both feature dimension reduction and feature selection are promising approaches to address this challenge, which can also solve over-fitting problem and reduce computational time. Feature selection is often useful to find discriminative and informative feature to obtain encouraging performance. For feature dimension reduction, widely used and effective methods are CCA, linear discriminant analysis (Polat et al., 2008), and principal component analysis (PCA). Essentially, these methods reduce dimension by mapping the feature into a low dimensional subspace with a transformation function, but they also suffer from limited interpretability. Another way to mitigate the dimension curse is to use popular feature encoding algorithm such as linear locally embedding (LLE; Shen et al., 2013), support vector coding (SVC; Yang et al., 2009), and the widely used bag of feature (BoF) representations (Stanciu et al., 2014) such as bag of visual word (BoVW; Fei-Fei and Perona, 2005; Lazebnik et al., 2006), vector locally aggregated descriptors (VLAD; Jégou et al., 2012; Li et al., 2014), and Fisher vector (FV; Sánchez et al., 2013; Lei et al., 2015c,d). These methods have shown compelling results in the computer vision field. Motivated by the promising performance of BoF, we use both the widely used BoVW and its variant VLAD for AD/MCI diagnosis and prognosis. Specifically, we investigate a BoF method to identify a particular stage between AD and healthy normal control (NC). Due to the variations of the multi-modal data in our database, a novel feature normalization method is developed to normalize the histogram feature obtained from the BoF pipeline. To the best of our knowledge, BoF has neither been used for AD/MCI diagnosis nor was associated with MRI/PET imaging data analysis.

In previous BoF pipeline, most work mainly concentrated on shallow structure, which is not desirable due to the ignorance of feature hierarchy. It has been shown that feature hierarchy by multilayer feature in the deep structure has achieved more encouraging results than that without feature hierarchy (Simonyan et al., 2013). Recently, spatial pyramid matching (SPM) strategy (Lazebnik et al., 2006) has shown its promising classification performance by exploring spatial information (i.e., pairwise and neighboring information) via the global representation. By partitioning the input signal uniformly into different regions and scales symmetrically, SPM has proved to be highly effective to improve descriptive power of the image representation and recognition accuracy. SPM method is furthered improved using linear sparse coding (Yang et al., 2009) and linear locality constrained coding (Wang et al., 2010). Recently, more advanced methods had been proposed to improve SPM further, e.g., VLAD (Jégou et al., 2012) and Fisher kernel encoding with Gaussian mixture model (GMM) based framework (Sánchez et al., 2013). Due to the powerful discriminative learning ability, deep learning has becoming a highly hot topic and gaining more and more popularity in the recent years, especially in the medical field (Chen et al., 2013). Actually, deep learning method with hierarchical feature design has achieved state-of-the-art performance in numerous tasks (Shin et al., 2013). For AD/MCI brain disease diagnosis, deep learning has been widely applied as well (Shin et al., 2013; Hjelm et al., 2014; Suk et al., 2014). For instance, Suk et al. (2014) use a high-level representation based on deep learning via restricted Boltzmann machine and deep Boltzmann machine to further improve performance. Inspired by this, we adopted a similar idea of deep learning method for the feature structure design. A feature hierarchy with multi-layer design is developed for the feature representation. Also, a novel feature normalization method is designed in order to reduce feature variations in different hierarchy.

Overall, we propose a novel framework for AD/MCI classification by learning discriminative features and fusing features of different modalities via CCA. The main contributions of our work are four-fold: (1) MRI and PET data are fused via CCA to make use of both individual and common features; (2) Deep feature architecture with multi-layer design and discriminative learning in feature encoding is investigated; (3) Different level of fusion method is developed to boost the diagnosis performance; (4) Novel feature normalization method is devised to improve the classification performance. Based on our extensive experiments on the ADNI dataset, our method achieves a classification accuracy of 96.93% (AD vs. NC), 86.75% (MCI vs. NC), and 82.75% [MCI converter (MCI-C) vs. MCI non-converter (MCI-NC)], which outperforms the state-of-the-art methods in the literature. The promising results validate the efficacy of CCA-based feature fusion and BoF-based feature representation for AD/MCI diagnosis.

## Materials and methods

### Materials and dataset

The publicly available ADNI dataset initialized by the National Institute on Aging (NIA), the National Institute of Biomedical Imaging and Bioengineering (NIBIB), the Food and Drug Administration (FDA), private pharmaceutical companies, and non-profit organizations has been utilized for performance evaluation. By utilizing MRI, PET, and other biomarkers, ADNI aims to facilitate the measurement of MCI progression and early AD. Until now, there are more than 800 senior adults participated this project. Specifically, an approximate of 200 cognitively normal older individuals follow up with the study for 3 years, and 400 people with MCI follow up for 2 years. Each local institutional review board approved the research protocol, and the written informed consent was obtained from each participant.

### Subjects

In this work, the pubic available ADNI dataset is utilized, but only the baseline MRI and 18-fluorodeoxyglucose PET (FDG-PET) data are used. There is a total of 93 AD subjects, 204 MCI subjects including 76 MCI converters (MCI-C), 128 MCI non-converters (MCI-NC), and 101 NC subjects. A detailed description of the clinic and demographic information is summarized in Table 1.

**Table 1:** **Clinical and demographic statistics (SD: standard deviation)**.

	**AD (93)**	**MCI(204)**	**NC(101)**
Female/Male	36/57	68/136	39/62
Age(mean ± SD)	75.49 ± 7.4	74.97 ± 7.2	75.93 ± 4.8
Age[min–max]	[55–88]	[55–89]	[62–87]
Education(mean ± SD)	14.66 ± 3.2	15.75 ± 2.9	15.83 ± 3.2
Education[min–max]	[4–20]	[7–20]	[7–20]
MMSE(mean ± SD)	23.45 ± 2.1	27.18 ± 1.7	28.93 ± 1.1
MMSE[min–max]	[18–27]	[24–30]	[25–30]
CDR(mean ± SD)	0.8 ± 0.25	0.5 ± 0.03	0 ± 0
CDR[min–max]	[0.5–1]	[0–0.5]	[0–0]

Among ADNI eligibility criteria (Alzheimer's Association, 2014), all subjects are aged from 55 to 90, an independent evaluation of functioning is conducted by study partner. The criteria (Suk et al., 2014) for general selection is as below: (1) NC subjects have MMSE scores ranging from 24 to 30 (inclusive), and a clinical dementia rating (CDR) of 0, which are non-depressed, non-MCI, and non-demented as well; (2) MCI subjects have MMSE scores ranging from 24 to 30 (inclusive), with complaint of memory loss. Objective memory loss measured by education adjusted scores on Wechsler Memory Scale Logical Memory, a CDR of 0.5, an absence of significant levels of impairment in other cognitive domains. MCI preserves daily living activities and dementia absence essentially. MMSE score of mild AD ranges between 20 and 26 (inclusive), and CDR of mild AD normally is 0.5 or 1.0. All the criteria satisfy the National Institute of Neurological and Communicative Disorders and Stroke and the Alzheimer's Disease and Related Disorders Association (NINCDS/ADRDA) criteria for probable AD.

### MRI/PET scanning and image processing

All structural MR images in this study were obtained from 1.5T scanners and downloaded in a Neuroimaging Informatics Technology Initiative (NIfTI) format. The preprocessing of these images includes spatial distortion correction caused by a gradient nonlinearity and B1 field inhomogeneity. The FDG-PET images were obtained 30–60 min post-injection, averaged, spatially aligned, interpolated to a standard voxel size, intensity normalization, rescaled to a common resolution of 8 mm, and with a full width at half maximum.

By applying the preprocessing such as the typical procedures of Anterior Commissure (AC)–Posterior Commissure (PC) correction, skull-stripping, and cerebellum removal, the MR images were preprocessed using MIPAV software 6 for AC–PC correction and resampled images to 256 × 256 × 256. N3 algorithm (Sled et al., 1998) is also applied to refine non-uniform tissue intensities. Skull stripping (Wang et al., 2014) and cerebellum removal are first applied, and the skull-stripped images are manually checked to ensure the clean and dura removal. FAST in FSL package 7 (Zhang et al., 2001) was employed to split the structural MR images into three tissue types of gray matter (GM), white matter (WM), and cerebrospinal fluid (CSF). Finally, all the three tissues were spatially normalized onto Kabani et al.'s atlas (Kabani et al., 1998). Although there are numerous advanced registration methods available for this process (Xue et al., 2006; Tang et al., 2009; Jia et al., 2010), the registration via HAMMER (Shen and Davatzikos, 2002) is selected and applied. Then, the regional volumetric maps named as RAVENS maps were produced by a tissue-preserving image warping method (Davatzikos et al., 2001). Given a quantitative representation of the spatial distribution of tissue types, the values of RAVENS maps are in a positive proportion of the amount of original tissue volume for each region. Since the high relation of AD/MCI and GM compared to WM and CSF (Liu et al., 2012), only the spatially normalized GM volumes (e.g., GM tissue densities) is considered for diagnosis in this work. Moreover, the FDG-PET images were rigidly aligned to the respective MR images. To improve signal-to-noise ratio, the GM density maps and PET images went through Gaussian smoothing further by a kernel method same as Liu M. et al. (2014), which shorten the computation time and reduces memory requirement without performance degradation.

### Methods

#### System overview

**Figure 1:**
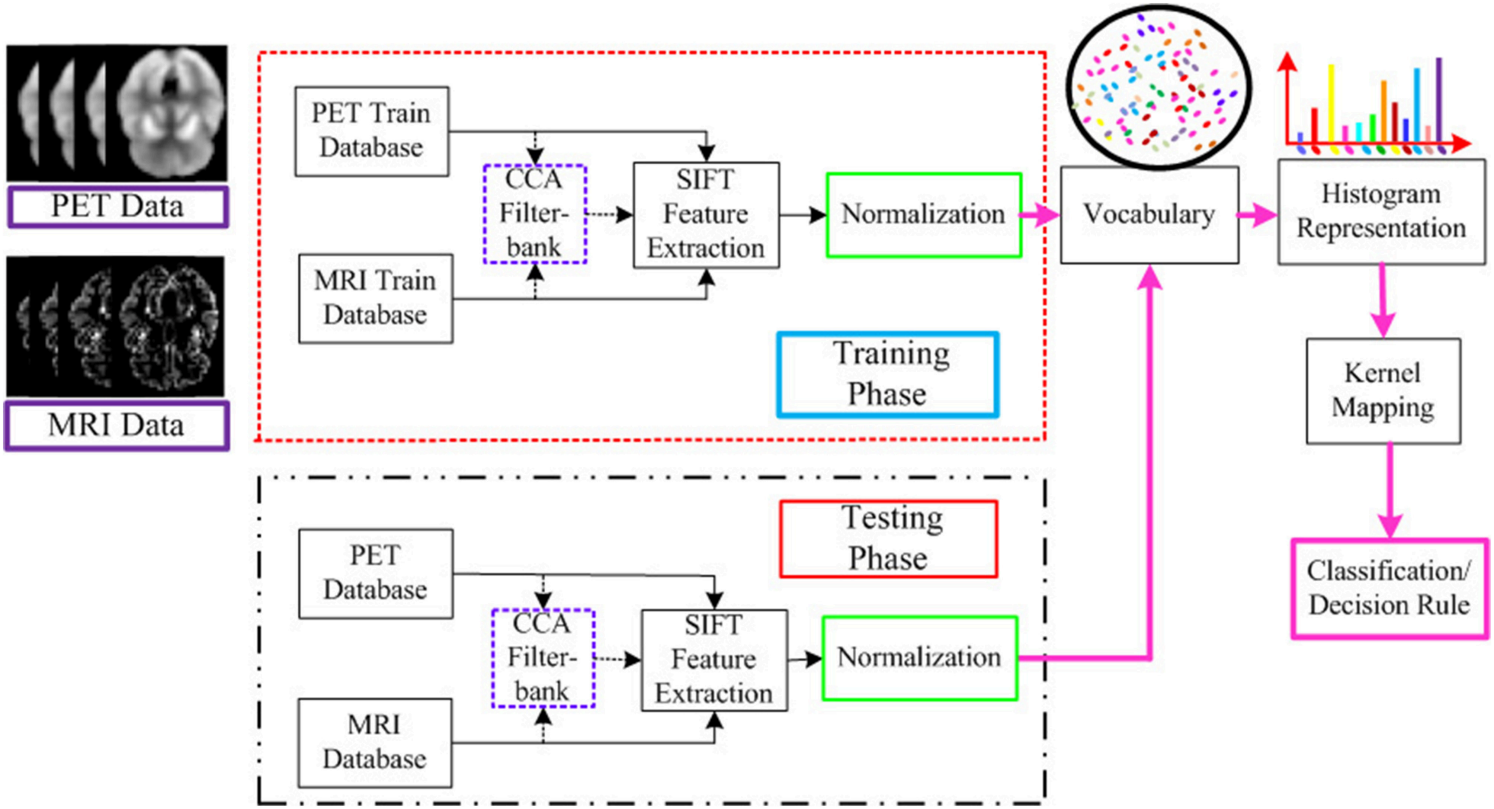
**Flowchart for the AD/MCI diagnosis system**. Feature extraction is based on both MRI and PET data. For the input MRI and PET data, CCA is first performed to extract the shared information between MRI and PET. And then the visual feature is extracted based on both the input MRI and PET data, and the fused data after CCA filterbank. A histogram representation is established based on the extracted feature by the standard BoF pipeline. The kernel mapping and normalization are applied to facilitate the classification. The final AD diagnosis results are based on the decision rule in the SVM classifier.

The flowchart for the AD/MCI diagnosis is illustrated in Figure 1. The system inputs are the preprocessed MRI and PET imaging data. The CCA filter-bank is applied to the input MRI and PET data to extract the common features of each sample between MRI and PET imaging data. For both MRI and PET modalities, a densely sampled SIFT descriptor (Vedaldi and Zisserman, 2012) is utilized to extract visual features from the input patches. As illustrated in the flowchart, the GM intensities of MRI and PET data are represented by component-wise dense SIFT descriptor. To enhance feature discriminability by exploring the intrinsic common feature, both MRI and PET modalities are fused with a canonical representation. Subsequently, we construct a histogram-based feature vector. Inspired by the explicit feature mapping using the non-linear kernel strategy in Vedaldi and Zisserman (2012), the kernel mapping method is applied in the diagnosis framework for performance boosting. After kernel mapping, the final histogram feature vectors are normalized and concatenated. Finally, support vector machine (SVM) classifier is utilized to classify the AD/MCI disease.

**Figure 2:**
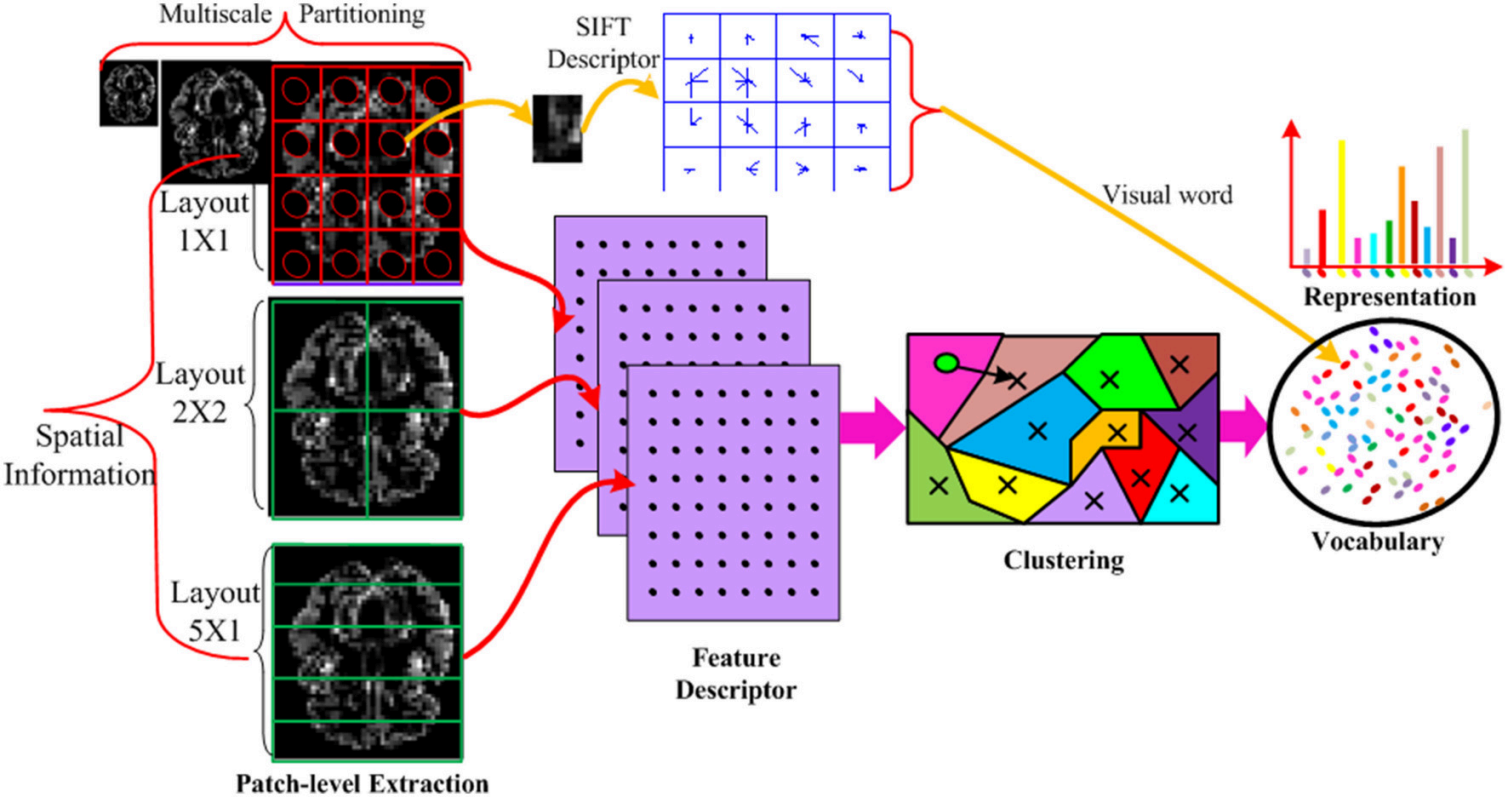
**Illustration of the feature vector formation using MRI imaging data only**. Each MRI imaging data is partitioned into different layouts using multi-scale techniques. In each patch, a SIFT feature is calculated and vector quantized into a visual word. The SIFT descriptor is extracted from all patches, all scales and all layouts. The descriptors from all the training data are mapped to visual words by *K*-means clustering technique to build a visual word vocabulary. The histogram of the BoVW representation is simply an occurrence count of the number of SIFT vectors assigned to each visual word. In the representation, the histogram has *K* bins generated by *K*-means. Each bin denotes an occurrence count of the SIFT vectors associated with that visual word. In this way, the imaging data is encoded into a feature vector.

The detailed procedure of the feature vector construction is illustrated in Figure 2. Specifically, the input MRI and PET data is partitioned into disjoint divisions at different scales to take advantage of spatial information and further increase the discriminability of the descriptor. In each division, the densely sampled SIFT feature (Vedaldi and Zisserman, 2012) is built. The extracted low-level dense SIFT features from each division is encoded to generate the visual words based vocabulary, for which we use the techniques of *K*-means clustering technique. An associated approximate nearest neighbor (ANN) determining the closest visual word to each descriptor based on a forest of randomized *K*-*d* tree is also recorded in the vocabulary (Muja and Lowe, 2014) to incorporate the neighboring information. This enables fast medium and large scale nearest neighbor queries among high dimensional data points (such as those produced by SIFT). The *K*-d tree data structure is able to quickly solve nearest-neighbor queries for the histogram representation. The entire histograms are concatenated from different divisions, and final concatenated histograms are constructed to form a long feature vector.

#### CCA

Feature fusion has been proved to be quite effective to greatly enhance the performance in a myriad of fields (Sun et al., 2013; Liu M. et al., 2014; Suk et al., 2014; Zhu et al., 2014a). In the AD/MCI diagnosis, MRI and PET modalities have played important roles as appearances, pattern, function, and structure are highly correlated with each other. With this high correlation, multimodal imaging feature fusion is able to enhance classification performance. It is known that CCA is a typical method to take the maximal advantage of the correlation among the multivariate random variables (Hardoon et al., 2004). Different from the previous study, CCA is investigated to identify the shared feature, and hence there is more information available in the feature subspace. By maximizing the correlation coefficient in a common space, fusing multimodal data by CCA can be used to extract the shared feature representation between MRI and PET (Zhu et al., 2014b).

Supposing that we have MRI and PET data (*d*-dimensional features from two different modalities of *n* samples) as follows:

(1) $$\begin{array}{r} {\text{X}}^{\left(1\right)}\in {\text{R}}^{d\times \text{n}},{\text{X}}^{\left(2\right)}\in {\text{R}}^{d\times \text{n}} \end{array}$$

(2) $$\begin{array}{r} \text{X}=\left[{\text{X}}^{\left(1\right)};{\text{X}}^{\left(2\right)}\right]\in {\text{R}}^{2d\times \text{n}} \end{array}$$

Where, the superscripts (1) and (2) denote MRI and PET, respectively. Given the covariance matrix of MRI and PET data is $\sum =\left(\begin{array}{cc} \Sigma_{11} & \Sigma_{12}\\ \Sigma_{21} & \Sigma_{22} \end{array}\right)$, CCA finds the projection matrices, B^(1)^ ∈ R^*d*×*d*^, B^(2)^ ∈ R^*d*×*d*^, by maximizing the correlation among features projected into the common space as follows:

(3) $$\left({\hat{\text{B}}}^{\left(1\right)},{\hat{\text{B}}}^{\left(2\right)}\right)=\arg\underset{\left({\text{B}}^{\left(1\right)},{\text{B}}^{\left(2\right)}\right)}{\max}\frac{{\text{B}}^{(1)\text{T}}\sum_{12}{\text{B}}^{\left(2\right)}}{\sqrt{{\text{B}}^{(1)\text{T}}\sum_{11}{\text{B}}^{\left(1\right)}}\sqrt{{\text{B}}^{(2)\text{T}}\sum_{22}{\text{B}}^{\left(2\right)}}}$$

This objective function can be solved by generalized eigen-decomposition. Thus, our canonical feature representation, Z^(1)^ and Z^(2)^, can be obtained by a weighted linear combination of the basis vectors as follows:

(4) $$\begin{array}{l} {\text{Z}}^{\left(1\right)}={\left({\hat{\text{B}}}^{\left(1\right)}\right)}^{T}{\text{X}}^{\left(1\right)},{\text{Z}}^{\left(2\right)}={\left({\hat{\text{B}}}^{\left(2\right)}\right)}^{T}{\text{X}}^{\left(1\right)} \end{array}$$

To increase discriminative ability, we combine the original features and the canonical feature representation together

(5) $$\begin{array}{l} \text{F}=\left[{\text{X}}^{\left(1\right)};{\text{X}}^{\left(2\right)};{\text{Z}}^{\left(1\right)};{\text{Z}}^{\left(2\right)}\right]\in {\text{R}}^{4d\times \text{n}} \end{array}$$

After constructing augmented feature vectors, the classification performance can be boosted with available auxiliary training data from the canonical projection (Shen et al., 2014). Namely, the augmented training data from other sources can provide better results compared to the original data if the extra and existing data have high correlation. By concatenating the MRI and PET multimodal information and canonical representations, the common and individual features are learned jointly, which benefits the AD/MCI diagnosis and prognosis.

#### Deep feature architecture

**Figure 3:**
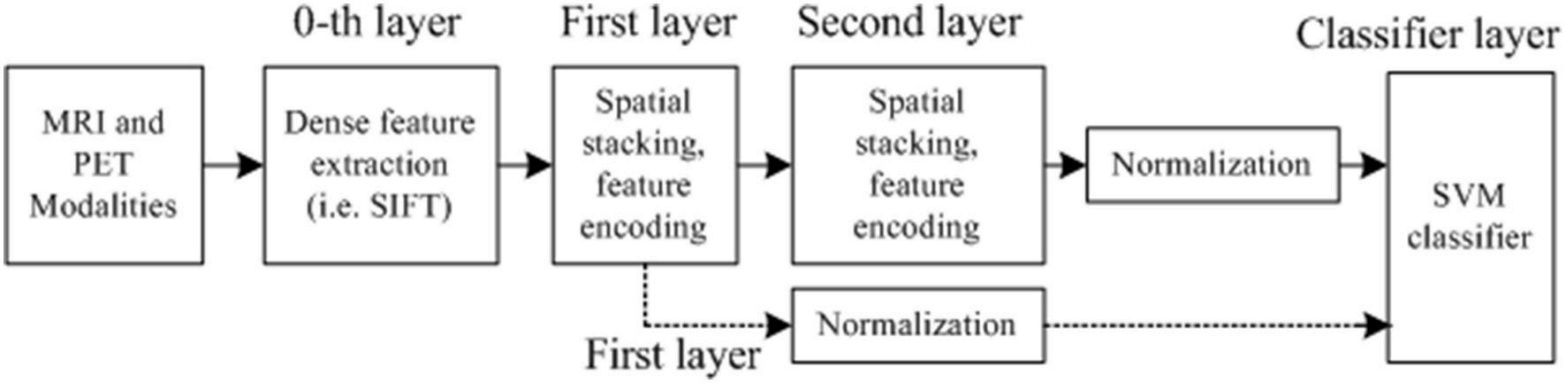
**The deep feature architecture for the AD diagnosis**.

Most of the previous studies focused on handcrafted features. However, this pipeline with a global feature vector is too shallow. As reported in Simonyan et al. (2013), feature hierarchy by multilayer feature is very effective to enhance the performance in that this structure builds the feature hierarchy for performance improvement. It is also demonstrated that deep learning representation with multilayer, e.g., convolutional neural network (CNN; Shin et al., 2013), delivers state-of-the-art performance in a myriad of classification tasks. In this regard, we design a deep multi-layer architecture for feature representation. Specifically, we first extract SIFT local features from the densely constructed patches as 0th layer. Since the low-level features are often indiscriminative and vulnerable to noises, they are mapped to middle-level features. As illustrated in Figure 3, we build a deep feature architecture to take advantage of the spatial information. Feature spatial layout strategy which divides an image into disjoint divisions (illustrated in feature formation steps in Figure 2) is also developed to explore the spatial information. Namely, the first layer only contains the feature extracted from all data without any partitioning. The second layer feature contains the disjoint subdivision information from the original modality, the deep feature architecture contains both first and second layer information from the original imaging modality and the subdivisions from the original imaging modality, whereas the first layer only contains the original imaging modalities information without any partitioning. By incorporating more spatial information, the deep feature architecture design has the capability to enhance the diagnosis performance.

#### Feature encoding

In our method, feature encoding is explored to map the extracted local features to codes. Compared with the local feature methods, the main advantage of encoding is that codes can be compared with simple Euclidean distance. Another advantage is that it is much easier to be learned by a classifier. Compared with feature selection methods (Chu et al., 2012; Zhu et al., 2014b), feature encoding gains an advantage by discriminative learning using high order statistics. Namely, feature selection only selects the discriminative or essential feature from the existing features, whereas feature encoding not only identifies the essential feature, but also incorporates high order statistics (e.g., co-covariance) and probability weights based on discriminative learning. The high order feature statistics provide auxiliary and useful information for classification, and high order statistics in feature encoding achieve better classification performance than that without them.

Before feature encoding, the clustering method is first applied to group the feature descriptors into different clustering centroid based on the similarity (Rodriguez and Laio, 2014). *K*-means (Leung and Malik, 2001) and spectral clustering (Ng et al., 2002) are popular clustering methods. Since *K*-means is probably one of the most popular ways for this task, it is selected in our method to construct codebook. Given a set of local features, *x*_1_, …, *x*_*m*_, …, *x*_*M*_, the objective of clustering is to find the representative *K* clusters, *d*_1_, …, *d*_*k*_, …, *d*_*K*_. For each feature *x*_*m*_, we define an indicator vector $\lambda_{m}\in {\left\{0,1\right\}}^{K}$ that indicates which cluster the feature vector belongs to. λ_*mk*_ becomes 1 if feature vector *x*_*m*_ is assigned to the *m*-th cluster, and 0 otherwise.

**Figure 4:**
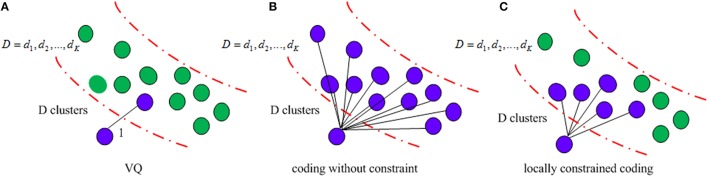
**Illustration of the clustering method (A) VQ; (B) coding without constraint; (C) locally constrained coding**.

It is known that the incorporating constraints into local structure increases robustness against noises (Yang et al., 2009). Figure 4 illustrates different structures used in clustering methods. VQ is the traditional method, which selects the nearest neighboring and encodes it as 1. The coding without any constraint clusters all the codes together, which is time consuming and indiscriminative. In contrast, the locally constrained coding only selects the *K* nearest neighbors and encodes them for learning. VLAD adopts the locally constrained coding strategy, which not only reduces computational time, but also increases discriminability as well.

Once a codebook learnt by *K*-means, μ_1_, …, μ_*m*_, …, μ_*M*_, we obtain a VLAD

1. Assign neighboring:
  (6) $$\text{NN}\left(x_{m}\right)=\arg{\text{min}}_{\mu_{k}}\left\|x_{m}-\mu_{k}\right\|$$
2. Compute *v*_*k*_:
  (7) $$\begin{array}{l} v_{k}=\sum_{x_{m}:\text{NN}\left(x_{m}\right)=\mu_{k}}x_{m}-\mu_{k} \end{array}$$
3. Concatenate *v*_*k*_ and normalize all feature vectors.

By concatenating the vectors together, we obtain a VLAD representation. The main purpose of encoding is to discriminate the distributional difference between a test image and all fitted training images. Essentially, BoVW is a simple counter of feature distribution and represented by the first moment information (i.e., cluster means), and the VLAD keeps both the first moment information and the residual information (i.e., mean and covariance of the distribution). One advantage of this is having the best representation of the feature descriptor distribution for discriminative classification. Another advantage is the alternative soft assignment of feature descriptor to the visual words is possible since the feature descriptor is distributed across several bins.

#### Feature normalization

In our dataset, testing and training data from different modality cause numerous variations in feature representation. A normalized feature representation helps improve classification accuracy (Sánchez et al., 2013) and hence feature normalization is first employed to lower the variance. *l*_*p*_-norm is a useful method to address the variation problem. After feature normalization, it is also shown that histogram normalization is especially beneficial for the SVM classifier (Vedaldi et al., 2009; Vedaldi and Zisserman, 2012). The widely applied normalization approaches include *l*_1_ normalization and *l*_2_ normalization. Since the large components can lead to suboptimal performance by affecting the SVM decision score with a dominating similarity, this effect should be suppressed. The signed square root normalization (a.k.a., power normalization) is used to suppress large components (Sánchez et al., 2013):

(8) $$\text{x }\leftarrow \;sign\text{​}\left(x\right)\text{​}{\left|x\right|}^{\rho },0\leq \rho \leq 1$$

where, ρ was set to 0.5 in our experiments, which is the same setting as that in Sánchez et al. (2013).

However, the drawback of traditional normalization method is the ignorance of the global and relational information among different subjects. To explore more relational information, a comprehensive normalization algorithm, namely, L2AL2W, is developed. This method makes use of the relation across and within subjects to enhance the AD/MCI diagnosis performance. Specifically, L2AL2W first performs *l*_2_ normalization for training and testing data of different subjects (across subject), and then *l*_2_ normalization is applied to the same feature vectors from the same subject (within subject). L2AL2W takes advantage of the relation between subject and feature, the decision boundary of the classifier is modified by the updated feature distribution, namely, the features are scaled based on both the subject information and feature information. The rationale behind the normalization method is that normalized histogram is distributed in the finite dimensions within [0,1]. By applying normalization across and within subjects, not only inter-subject variations are reduced, intra-subjects variations are probably expanded. Accordingly, competing classification results are obtained by affecting decision boundary of SVM classifier.

#### Fusion strategy

**Figure 5:**
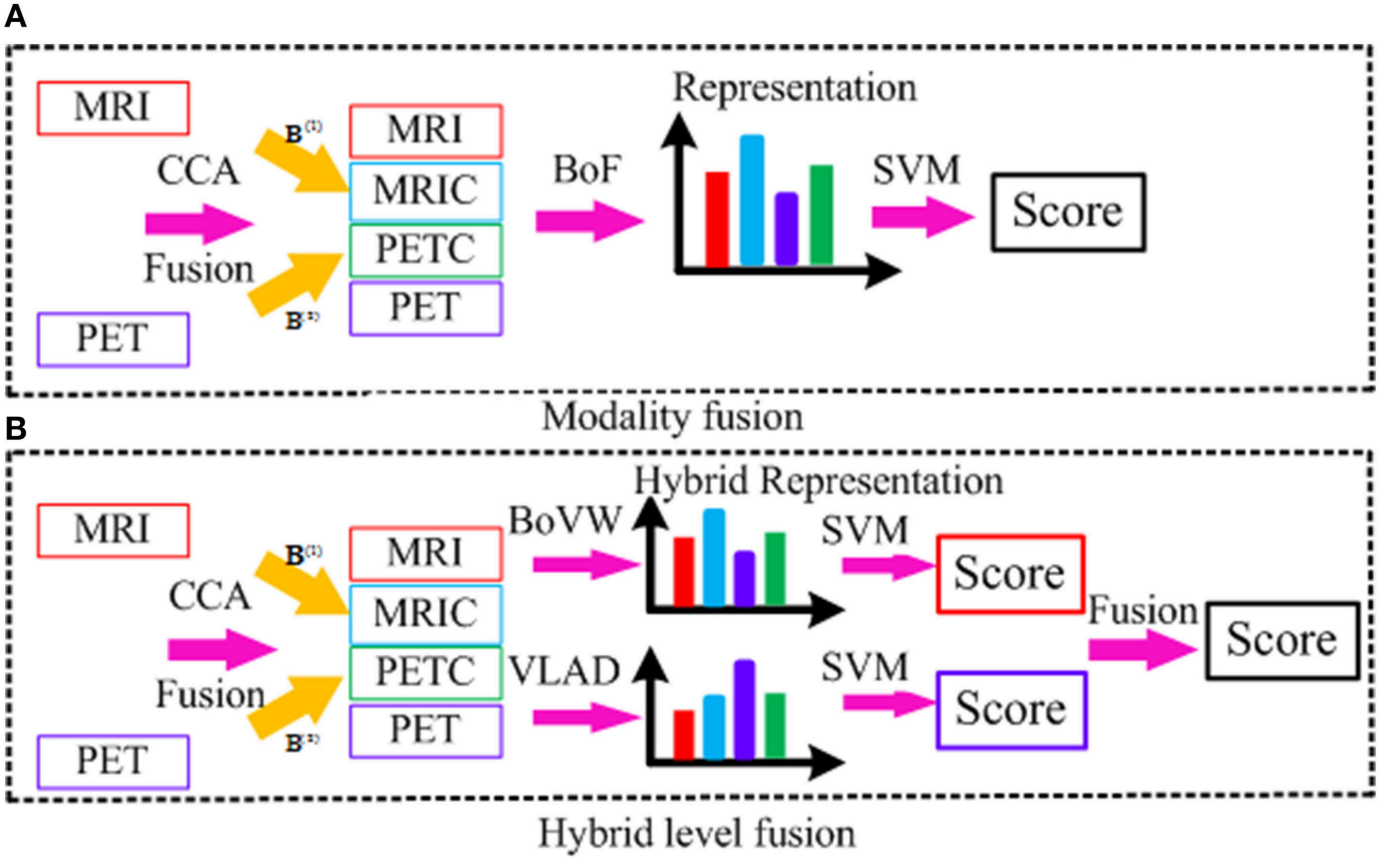
**Illustration of different fusion methods; (A) modality fusion; (B) hybrid level fusion**.

Figure 5 illustrates both low-level modality fusion and hybrid level fusion. Low-level modality fusion involves concatenation of multiple modalities and their corresponding CCA projections. That is, MRI and projected MRI via CCA (MRIC), PET and projected PET via CCA (PETC), are concatenated together. For hybrid representation, the widely applied encoding methods such as BoVW and VLAD are explored. Initially proposed by Jégou et al. (2012), VLAD is an improved version of BoVW obtained by aggregating BoVW representation with high order statistics. Both BoVW and VLAD representation are promising ways for classifying patterns distinctively via discriminative learning. To make use of both, we propose to combine them at the classifier-level. Let *S*_*BoVW*_ and *S*_*VLAD*_ denote the scores from the two classifiers, to which BoVW and VLAD representations are fed into as inputs, respectively. We then combine the scores as follows:

(9) $$\begin{array}{l} {S=\alpha S}_{BoVW}+\left(1-\alpha \right)S_{VLAD} \end{array}$$

where, α is a fusion weight. The weight is chosen adaptively based on cross-validation.

The motivation behind the hybrid level fusion is that low-level fusion is effective when MRI and PET modality is highly correlated, whereas the score level fusion is effective when each subject is independent with each other. However, each modality is neither fully correlated nor each subject is fully independent, and the simple fusion method may not achieve a satisfying result. Hybrid fusion takes advantage of both BoVW and VLAD representation together, which is able to boost the diagnosis performance with feature complementarities in the standard BoF pipeline. This simple yet effective feature representation benefits from exploration of different encoding methods, and thus the state-of-the-art performance can be obtained.

## Experimental result

### Experiment setup

Both the individual feature and shared feature fusion by CCA is evaluated in our experiments to validate the effectiveness of three binary classifiers for AD vs. NC, MCI vs. NC, MCI converter (MCI-C) vs. MCI non-converter (MCI-NC). The performance evaluation is based on 10-fold cross validation, that is, each dataset is randomly partitioned into 10 subsets, where 10 and 90% is used for testing and training, respectively. Each experiment is repeated 10 times to remove the introduced bias. A linear SVM classifier is utilized for the performance evaluation. The methods in Liu M. et al. (2014) and Suk et al. (2014) are selected for performance comparison based on the same dataset since they are similar. Same as Suk et al. (2014), the diagnostic performance is quantitatively evaluated by the recognition accuracy (the disease status of subjects is correctly classified as the actual disease status of the subjects in each class) (ACC), sensitivity (SEN), specificity (SPEC), balanced accuracy (BAC), positive predicted value (PPV), negative predictive value (NPV), area under the receiver operating characteristic curve (AUC) is also utilized for the evaluation metrics. The same definition of the quantitative measurements as Suk et al. (2014) are utilized to evaluate the diagnosis performance, which are denoted as below:

- ACC = (TP+TN)/(TP+TN+FP+FN)
- SEN = TP/(TP+FN)
- SPEC = TN/(TN+FP)
- BAC = (SEN+SPEC)/2
- PPV = TP/(TP+FP)
- NPV = TN/(TN+FN)

where TP, TN, FP, FN are true positive, true negative, false positive, and false negative, respectively.

### Diagnosis results

Table 2 summarizes the classification results for AD/MCI diagnosis and prognosis, where MRIPET means the simple concatenation of MRI and PET, and MRIPETHF means the hybrid level fusion of MRI and PET. The performance comparison of both Liu et al.'s and Suk et al.'s methods are also summarized in Table 2. For the AD and NC classification, compared with Suk et al.'s Suk et al. (2014) and Liu et al.'s Liu M. et al. (2014) methods, the proposed method exhibited maximal accuracies of 96.93% (AD vs. NC), 86.57% (MCI vs. NC), and 82.75% (MCI-C vs. MCI-NC), respectively. The accuracy of the proposed method is slightly higher than Suk et al.'s method, but it shows a significant improvement over Liu et al.'s method. Specifically, compared with the best performance of Suk et al.'s method, the proposed method shows a performance improvement of 1.66% (AD vs. NC), 1.05% (MCI vs. NC), and 8.34% (MCI-C vs. MCI-NC). Discrimination between MCI-C and MCI-NC are the most challenging for early treatment and diagnosis. For MCI-C and MCI-NC classification, our method has an improvement of 10.63% (MRI), 12.96% (PET), 8.34% (MRI+PET), respectively, over Suk et al.'s method.

**Table 2:** **Diagnosis results**.

	**Method**	**Modality**	**ACC(%)**	**SEN(%)**	**SPEC(%)**	**BAC(%)**	**PPV(%)**	**NPV(%)**	**AUC(%)**
AD/NC	Liu M. et al., 2014	MRI	90.18 ± 5.25	91.54	90.61	90.67	88.94	90.67	96.20
		PET	89.13 ± 6.81	90.06	89.36	89.71	88.49	89.26	95.94
		MRIPET	90.27 ± 7.02	89.48	92.44	90.96	90.56	88.7	96.55
	Suk et al., 2014	MRI	92.38 ± 5.32	91.54	94.56	93.05	92.65	90.84	96.97
		PET	92.20 ± 6.70	88.04	96.33	92.19	95.03	89.66	97.98
		MRIPET	95.35 ± 5.23	94.65	95.22	94.93	96.80	95.67	98.77
	Proposed	MRI	91.76 ± 6.14	91.01	91.54	92.44	92.17	90.86	92.44
		PET	90.89 ± 5.81	93.32	88.01	91.53	88.96	91.32	91.53
		MRIPET	94.4 ± 5.65	97.35	90.79	95.03	91.72	93.25	95.03
		MRIPETHF	96.93 ± 2.65	97.48	93.65	96.41	99.09	94.16	95.7
MCI/NC	Liu M. et al., 2014	MRI	81.00 ± 4.98	97.08	48.18	72.63	79.14	88.99	72.63
		PET	81.14 ± 10.22	96.03	52.59	74.31	80.26	84.16	74.31
		MRIPET	83.9 ± 5.80	98.97	52.59	75.78	81.18	97.22	75.78
	Suk et al., 2014	MRI	84.24 ± 6.26	99.58	53.79	76.69	81.23	98.75	76.69
		PET	84.29 ± 7.22	98.69	56.87	77.78	81.99	94.57	77.78
		MRIPET	85.67 ± 5.22	95.37	65.87	80.62	85.02	89.00	80.62
	Proposed	MRI	83.52 ± 5.38	92.07	50.28	84.98	79.99	88.75	84.98
		PET	82.95 ± 6.37	91.78	50.64	77.25	81.09	90.43	77.25
		MRIPET	83.67 ± 5.49	91.03	51.12	78.39	82.48	91.23	78.39
		MRIPETHF	86.57 ± 4.72	95.41	52.79	82.03	81.26	92.42	82.03
MCI-C/MCI-NC	Liu M. et al., 2014	MRI	64.75 ± 14.83	22.22	89.57	55.90	46.29	77.39	55.90
		PET	67.17 ± 13.43	40.02	82.61	61.32	64.13	70.31	61.32
		MRIPET	73.33 ± 12.47	33.25	97.52	65.38	80.00	73.18	65.38
	Suk et al., 2014	MRI	72.42 ± 13.09	36.70	90.98	55.90	46.29	77.84	55.90
		PET	70.75 ± 13.23	25.45	96.55	61.32	64.13	70.69	61.32
		MRIPET	75.92 ± 15.37	48.04	95.23	65.38	80.00	74.33	65.38
	Proposed	MRI	80.12 ± 8.65	62.42	89.06	81.52	82.14	84.55	81.52
		PET	79.92 ± 4.72	59.37	91.12	82.45	85.14	86.98	82.45
		MRIPET	81.57 ± 8.66	66.52	89.96	79.67	83.41	88.92	79.67
		MRIPETHF	82.75 ± 4.81	77.29	95.08	85.27	90.36	90.23	85.27

Probability of misdiagnosing AD/MCI patients reduces with increasing sensitivity, and probability of misdiagnosing NC as AD/MCI reduces with increasing specificity. It can be seen that the proposed method has a lower performance than Suk et al.'s method in some cases in terms of sensitivity and specificity. It is obvious that our method outperforms Liu et al.'s method significantly especially for the MCI-C and MCI-NC classification.

Based on the PPV and NPV results for AD, MCI and MCI-NC, it can be observed that PPV and NPV results are quite good. That is, the high percentage of AD, MCI, or MCI-NC subjects can be diagnosed correctly. From the above-mentioned quantitative measurements, it can be shown that our method outperforms the competing methods in most cases. The main reason is that multi-modality data and fusion improve the classification performance significantly.

Due to imbalance between classes [i.e., AD (93 subjects), MCI (204 subjects; 76 MCI-C and 128 MCI-NC subjects), and NC (101 subjects)], low sensitivity (MCI vs. NC) or specificity (MCI-C vs. MCI-NC) is obtained. The balanced accuracy is calculated to avoid inflated performance estimates on imbalanced datasets. From BAC result, it is clear that the proposed method performs better than Liu et al.'s and Suk et al.'s methods.

Unlike hierarchical fusion method (Liu M. et al., 2014) and traditional methods of concatenating features from multiple modalities into a long vector (Suk et al., 2014; Zhu et al., 2014a), both CCA and discriminative learning are investigated to fuse multimodal data with consideration of common and essential features from different feature spaces (Hardoon et al., 2004; Shen et al., 2014).

### Effect of different modalities

**Figure 6:**
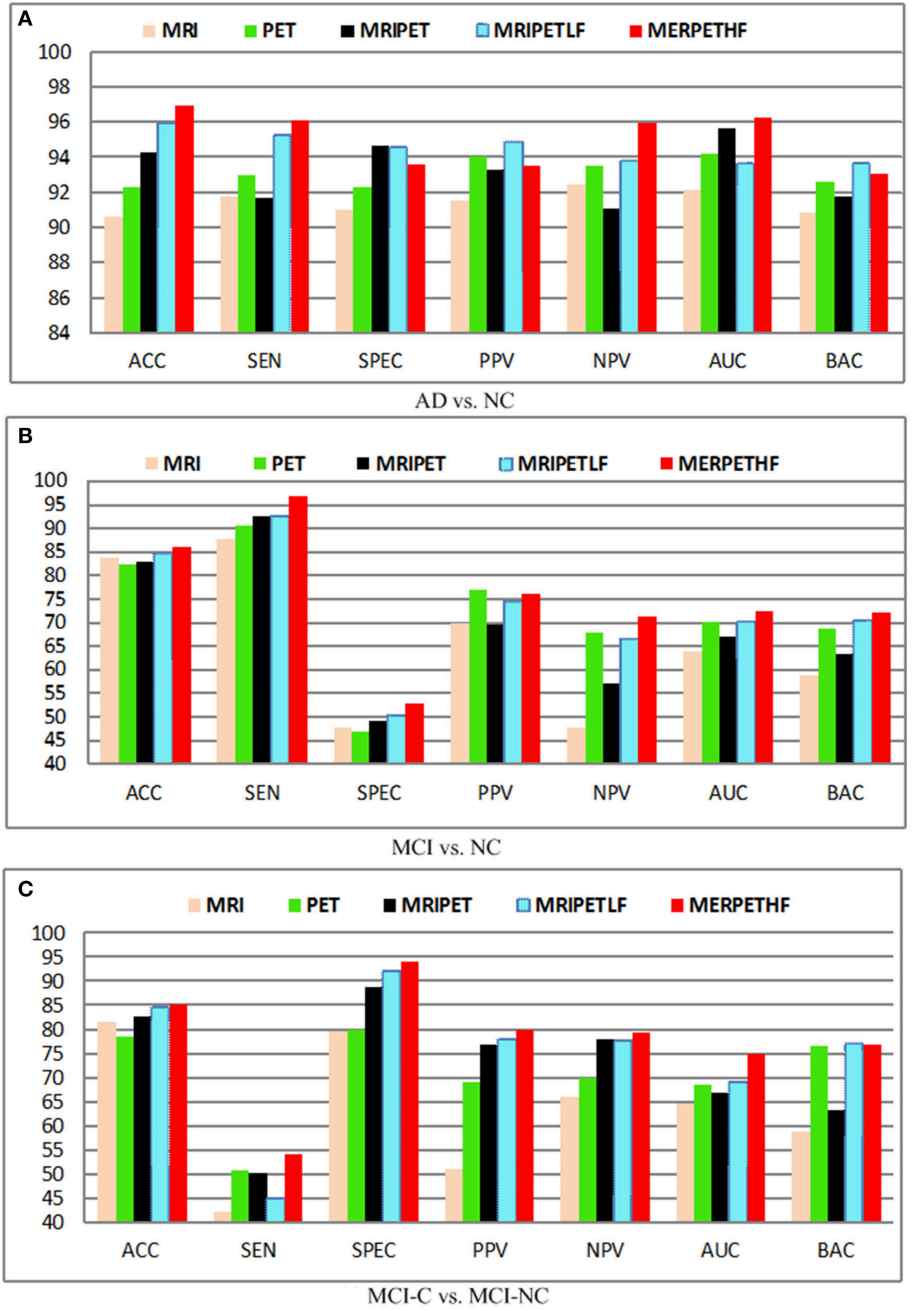
**Diagnosis results with different modalities. (A)** AD vs. NC; **(B)** MCI vs. NC; **(C)** MCI-C vs. MCI-NC.

Figure 6 shows the diagnosis results with different fusion method, where MRIPET means simple concatenation, MRIPETLF means the modality level fusion of MRIPET, and MRIPETHF means hybrid level fusion including both modality fusion and score fusion. It is known that the increased in performance by modality fusion is mainly due to the feature complementarity. The complementarity is not limited to the exploration of different modality, which can be further extended into different encoding methods. Since VLAD extends BoVW's zero statistics by introducing high order statistics, these two encoding methods have essential complementary information for each other. Better performance can be obtained by the hybrid representation than simple representation. It can be seen that hybrid fusion is the best choice for all scenarios, which indicates that multiple descriptors are highly correlated in the modality level. In general, fusion method is quite effective to improve the performance, and hybrid level fusion generally outperforms the simple concatenation. Based on the above observations and analysis, we can conclude that fusion method is able to boost AD/MCI diagnosis performance.

### Effect of feature hierarchy

**Figure 7:**
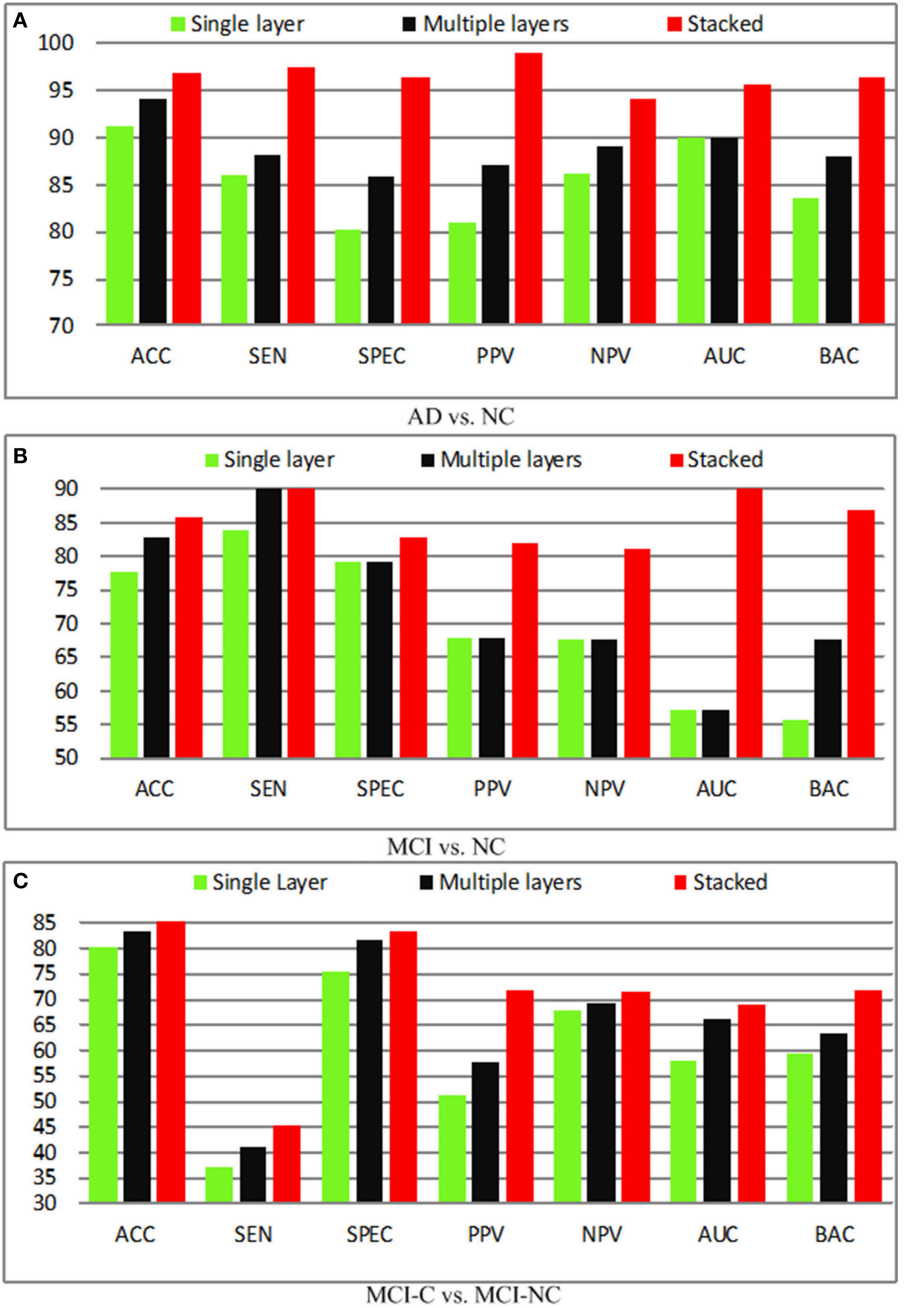
**Diagnosis results with different layers. (A)** AD vs. NC; **(B)** MCI vs. NC; **(C)** MCI-C vs. MCI-NC.

It is known that a single layer is not sufficient to improve the performance due to its shallow representation, and hence feature hierarchy based on multiple layers is utilized. Besides, the combination of the single layer and multiple layers (stacked) will further boost the performance. In this section, we compare the performance of the single layer, multiple layers, as well as the stacked representation. The quantitative analysis of different layers in the feature representation is performed and demonstrated in Figure 7. In the baseline, the shallow representation with a single layer achieves accuracies of 91.32, 77.67, 80.25%, respectively. A significant performance boosting has achieved by injecting a single intermediate layer, which leads to 3.15% improvement in terms of the accuracy, whereas the stacked representation further improves accuracy by 8.34%. The encouraging results obtained by adding a single intermediate layer based on similar deep learning architecture. In fact, the state-of-the-art performance is achieved by stacked representation in our proposed method.

**Figure 8:**
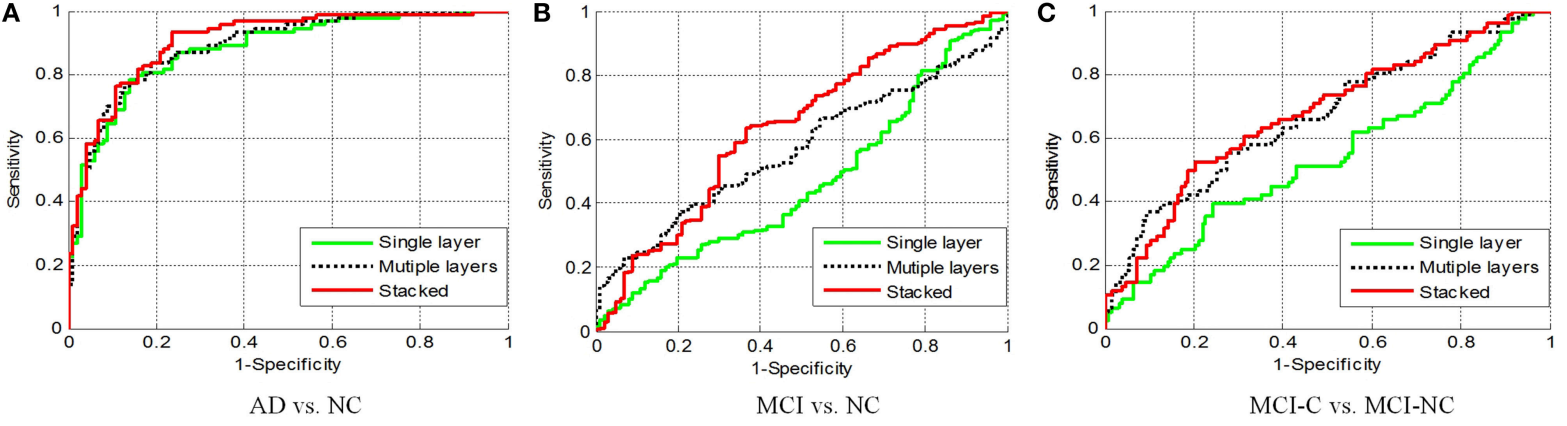
**RoC results with different layers. (A)** AD vs. NC; **(B)** MCI vs. NC; **(C)** MCI-C vs. MCI-NC.

Figure 8 shows the receiver operating characteristic (RoC) curves of the proposed method based on the single layer, multiple layers, and stacked representation. It is obvious that the proposed hybrid fusion achieves the best performance in all experiments.

### Effect of different normalized methods

**Figure 9:**
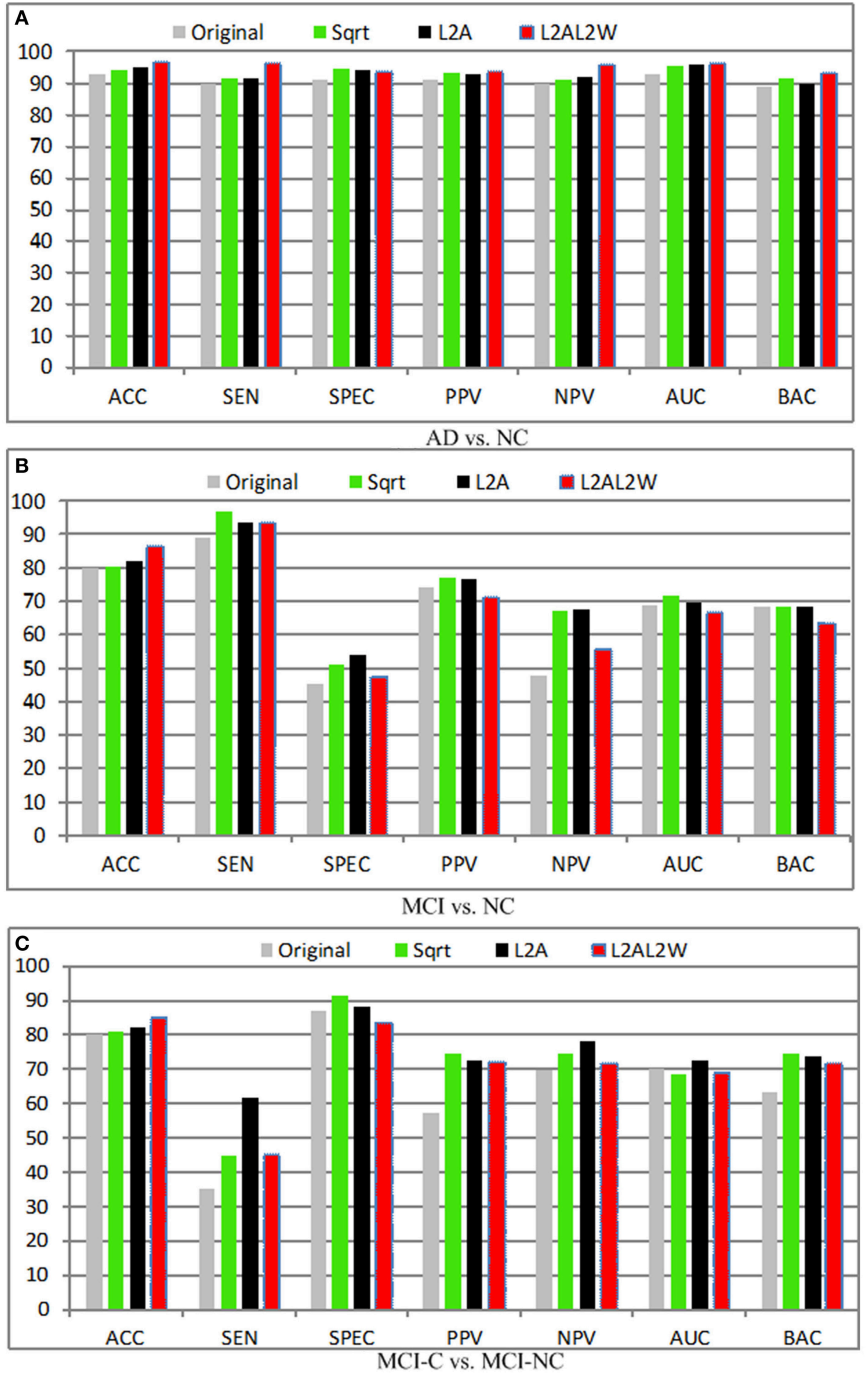
**Diagnosis results with different normalization methods. (A)** AD vs. NC; **(B)** MCI vs. NC; **(C)** MCI-C vs. MCI-NC.

Apart from conventional normalization methods, novel normalization methods are proposed, and evaluated for super vector based encoding methods. *l*_2_ Normalization generally outperforms *l*_1_ normalization when the kernels are utilized in the classifier. The main explanation is: if K(x, y) = x^*T*^y is interpreted as a distance score nearest to itself, when the kernel K(x, y) is used in the linear SVM classifier, *l*_2_ normalization can guarantee: K(x, y) = *C* and K(x, x) > K(x, y), and hence a simple consistency criterion is achieved. However, the choice of *l*_1_-normalization is unable to ensure this criterion and leads to instability in the SVM training. In view of this, only *l*_2_ normalization is compared and reported in this section. Figure 9 illustrates the experimental results in terms of different normalization methods, where Sqrt denotes the power normalization, L2A means L2 normalization across the subjects, and L2AL2W represents the inter- and intra-subject normalization. Several observations can be concluded from these results:

1. For the support vector based encoding methods, normalization is a promising way to reduce variations and make maximal use of the relational information across and within subjects.
2. The feature bursts of the background can be suppressed by the power normalization method. By removing the bursting effect, the power normalization is especially effective to boost the super-vector based representation using the sum pooling.
3. Generally, *l*_2_ normalization achieves good performance with the linear SVM.

### Classification performance using kernel mapping

As shown in Vedaldi and Zisserman (2012), the classification performance can be boosted by kernel mapping, which approximate the feature map via non-linear kernel. The motivation is to use non-linear mapping for the linear SVM method to reduce the computation time. This kernel trick can be applied for distance metric based algorithms. The classification algorithm based on inner product (i.e., linear SVM) is computed in a non-linear way via inner product with a suitable kernel or feature map. For example, feature map for Hellinger (or Bhattacharyya) kernel is obtained by calculating the element-wise square root. It is shown that the Hellinger kernel is equal to the Euclidean distance in feature map. Hellinger kernel is demonstrated to produce superior results than a linear kernel.

**Figure 10:**
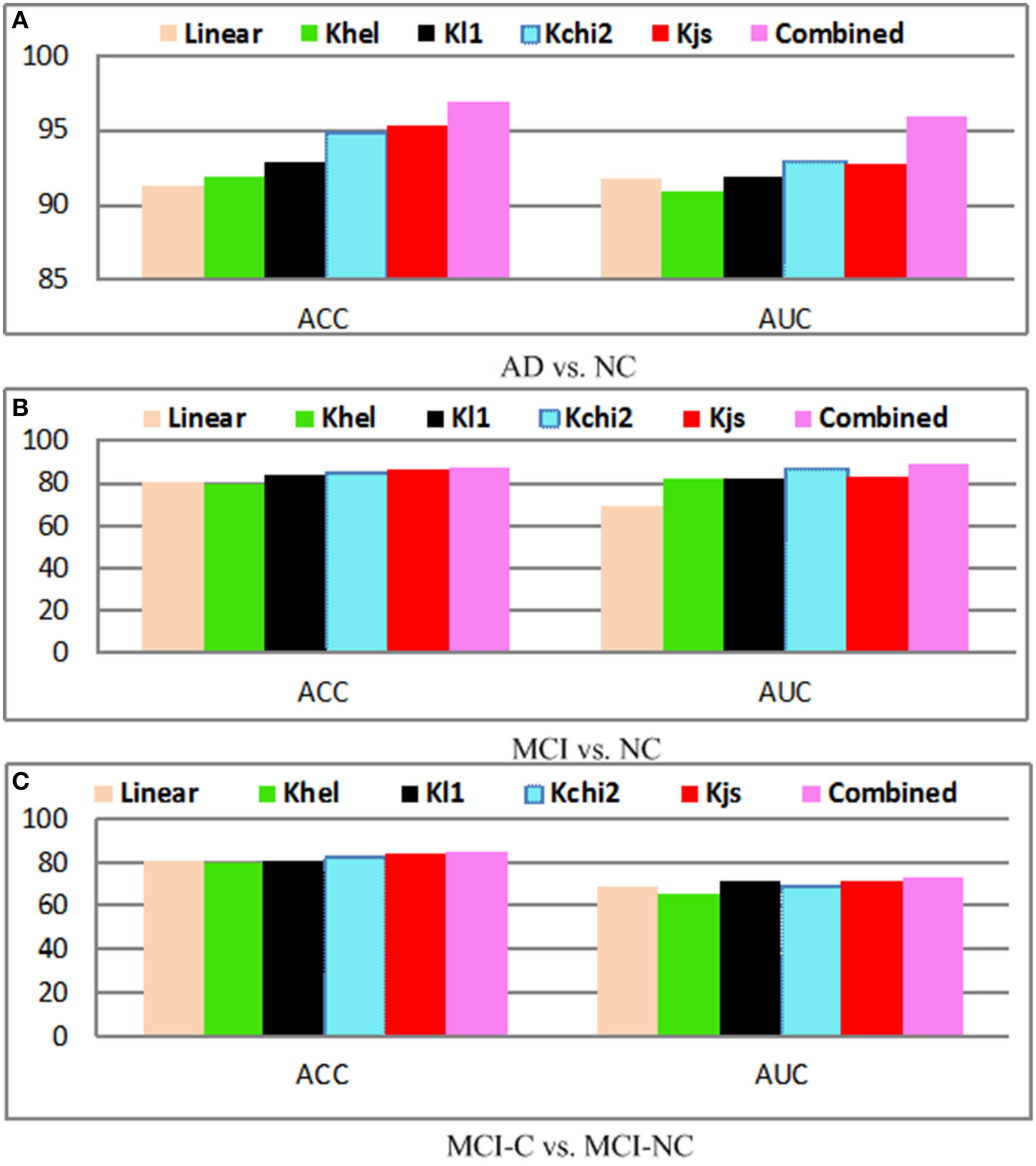
**Diagnosis results with different kernels; (A) AD vs. NC; (B) MCI vs. NC; (C) MCI-C vs. MCI-NC**.

The performance with and without kernel mapping of our proposed method is shown in Figure 10. The linear algorithm (Linear) and nonlinear mapping kernels including chi2-square kernel (Kchi2), Jensen-Shannon kernel (Kjs) are evaluated. The optimal parameters for kernel mapping are selected via grid search. Generally, the approach without kernel obtains the worst performance for AD vs. NC, MCI vs. NC, and MCI-C vs. MCI-NC classification results, which justifies the significance of the kernel mapping for the disease diagnosis. It can be observed that Kjs kernel achieves superior performance among different kernels, whereas the combined method achieves the highest performance. Generally, kernel based mapping outperforms the nonlinear method, and combined method achieves the state-of-the-arts performance.

### Comparison with state-of-the-art methods

The diagnosis performance is also compared with the state-of-the-art algorithms with and without multi-modality information in the widely applied AD vs. NC, and MCI-C vs. MCI-NC classification tasks. Since different datasets and approaches may have different features and classifiers, direct fair comparison is difficult or impossible. Nevertheless, the achieved accuracy in the classification problems shows the effectiveness of the proposed method. Tables 3, 4 summarized comparisons of different algorithms for AD vs. NC and MCI-C vs. MCI-NC classification, respectively. It can be seen that the fusion methods are generally quite effective for the AD/MCI diagnosis. Multimodality and multi-atlas are also able to enhance the classification performance than the unimodal and single atlas methods.

**Table 3:** **Algorithm comparison for AD vs. NC classification results**.

**Algorithm**	**Subject**	**Modality**	**AD vs. NC (%)**
Zhang and Shen, 2011	51AD+99MCI+52NC	PET+MRI	90.6
Zhang and Shen, 2011	51AD+99MCI+52NC	PET+MRI+CSF	93.2
Hinrichs et al., 2011	48AD+66NC	PET+MRI	87.6
Hinrichs et al., 2011	48AD+66NC	PET+MRI+CSF+APOE+cognitive scores	92.4
Kohannim et al., 2010	88AD+115NC	Multi-atlas	86.0
Liu et al., 2012	198AD+229NC	Single atlas	90.8
Gray et al., 2013	51AD+99MCI+52NC	PET+MRI	94.37
Liu et al., 2014	51AD+99MCI+52NC		92.82
Min et al., 2014	91AD+128NC	Data-driven GM ROI	91.64
Zhu et al., 2014a	51AD+99MCI+52NC	PET+MRI+CSF	95.9
Suk et al., 2014	128AD+169MCI+101NC	MRI+PET	95.35
Proposed	128AD+169MCI+101NC	MRI+PET	96.93

**Table 4:** **Algorithm comparison for MCI-C vs. MCI-NC classification results**.

**Algorithm**	**Subject**	**Modality**	**MCI vs. NC (%)**
Davatzikos et al., 2010	69MCI-C+170 MCI-NC	CSF+MRI	61.7
Cuingnet et al., 2011	76MCI-C+134MCI-NC	Single-atlas	70.4
Zhang and Shen, 2011	38MCI-C+50MCI-NC	PET+MRI +CSF+APOE	78.4
Coupé et al., 2012	167MCI-C+238MCI-NC	MRI	71.0
Kohannim et al., 2010	54MCI-C+115MCI-NC	Multi-atlas	72.1
Young et al., 2013	47MCI-C+96MCI-NC	PET+MRI	74.1
Min et al., 2014	117MCI-C+117MCI-NC	Multi-atlas	72.41
Zhu et al., 2014a	51AD+99MCI+52NC	PET+MRI CSF	72.6
Suk et al., 2014	128MCI-C+76MCI-NC	PET+MRI	75.42
Proposed	128MCI-C+76MCI-NC	PET+MRI	82.75

## Discussions

Although efficacy is achieved in our experiments for the three classification problems, there are still some limitations of the proposed method. First, it is quite difficult to interpret the brain and neurodegenerative disease (i.e., AD or MCI) using feature representation in the clinical application. There is not sufficient clinical information to find the brain ROI regions for the clinical understanding of the brain abnormalities. Second, the parameters for feature extraction, clustering and coding may not be optimally determined. Intensive study is also required to find the optimal parameter via optimization method. Third, only MRI and PET modalities are explored in this study, more auxiliary information such as CSF and clinical information may be beneficial for the AD/MCI if they have high correlations. Also, there is other information available such as cognitive, genetics, proteomics, and psychological perspective, which may further improve the performance. Fourth, modality level fusion emphasizes the dependence in imaging approaches, high dimensional features in the codebook training, which causes the instability in the unsupervised training. Finally, discriminative feature encoding methods are able to achieve very promising performance with histogram level representation, but the final dimension of combined representation is large. High dimension may render the SVM classifier to be computational intensive, and the codebook size of the super vector generated from the encoding algorithm is too small. Meanwhile, there are some suggestions to address these limitations. The fusion different feature representation could be an effective way to interpret AD disease. The optimal parameters can be obtained by inner cross validation or evolutional algorithm such as genetic algorithm. However, it takes a long time to compute the best parameters. More modality should be considered to boost the performance. Lastly, high dimension problem can be addressed by feature dimensionality reduction algorithm.

Overall, we can have the following findings summarized based on the extensive experiments. First, transforming the local feature from descriptor to codeword space and from 0-th statistics to high order statistics is able to boost the classification performance (Sánchez et al., 2013). Second, fusion of different level of modalities and hybrid level representation are able to improve the performance greatly. Third, feature normalization is often ignored in previous studies. Novel intra- and inter-normalization strategies are developed instead of using the previous normalization method. This novel feature normalization has the capability to reduce the variations and makes use of the relational data as well due to a large number of the unrelated feature. Feature normalization is highly important to boost the performance. Last but not least, it is important to preprocess the data to enhance the diagnosis performance. Generally, discriminative learning based representation is quite effective and efficient to encode data and obtain promising results. The sparse feature is not stable and reliable enough to achieve encouraging AD/MCI results. Hard assignment may not have discriminability power for the code words, whereas the soft assignment is able to further distinguish the neuro-disease by the enhanced discriminative power of the generated visual words.

## Conclusions

In this paper, a fusion method via shared and individual feature representation based on MRI and PET is proposed for AD/MCI diagnosis. Different from the unimodal method using GM intensities only, both MRI and PET features are fused together to incorporate the complementary feature. CCA is employed to identify the latent and joint feature from multi-modality in the feature representation. The complementary feature from MRI and PET can boost the performance. Novel normalization method is designed to further improve the performance. It is found that kernel technique with feature hierarchy could further improve the performance. Extensive experimental on publicly available ADNI dataset demonstrated that the proposed method outperforms related methods based on quantitative measurements.

## Author contributions

BL wrote the main manuscript text. All authors reviewed the manuscript.

### Conflict of interest statement

The authors declare that the research was conducted in the absence of any commercial or financial relationships that could be construed as a potential conflict of interest.
